# Endogenous Fructose Metabolism Could Explain the Warburg Effect and the Protection of SGLT2 Inhibitors in Chronic Kidney Disease

**DOI:** 10.3389/fimmu.2021.694457

**Published:** 2021-06-16

**Authors:** Takahiko Nakagawa, Laura G. Sanchez-Lozada, Ana Andres-Hernando, Hideto Kojima, Masato Kasahara, Bernardo Rodriguez-Iturbe, Petter Bjornstad, Miguel A. Lanaspa, Richard J. Johnson

**Affiliations:** ^1^ Department of Nephrology, Rakuwakai Otowa Hospital, Kyoto, Japan; ^2^ Department of Biochemistry, Shiga University of Medical Science, Otsu, Japan; ^3^ Department of Cardio-Renal Physiopathology, Instituto Nacional de Cardiología Ignacio Chavez, Mexico City, Mexico; ^4^ Division of Renal Diseases and Hypertension, University of Colorado Denver, Aurora, CO, United States; ^5^ Institute for Clinical and Translational Science, Nara Medical University Hospital, Kashihara, Japan; ^6^ Department of Nephrology, Instituto Nacional de Ciencias Medicas y Nutricion Salvador Zubiran and Instituto Nacional de Cardiologia Ignacio Chavez, Mexico City, Mexico; ^7^ Department of Pediatrics-Endocrinology, University of Colorado Denver, Aurora, CO, United States

**Keywords:** fructose, The Warburg effect, CKD - chronic kidney disease, inflammation, fibrosis

## Abstract

Chronic low-grade inflammation underlies the pathogenesis of non-communicable diseases, including chronic kidney diseases (CKD). Inflammation is a biologically active process accompanied with biochemical changes involving energy, amino acid, lipid and nucleotides. Recently, glycolysis has been observed to be increased in several inflammatory disorders, including several types of kidney disease. However, the factors initiating glycolysis remains unclear. Added sugars containing fructose are present in nearly 70 percent of processed foods and have been implicated in the etiology of many non-communicable diseases. In the kidney, fructose is transported into the proximal tubules *via* several transporters to mediate pathophysiological processes. Fructose can be generated in the kidney during glucose reabsorption (such as in diabetes) as well as from intra-renal hypoxia that occurs in CKD. Fructose metabolism also provides biosynthetic precursors for inflammation by switching the intracellular metabolic profile from mitochondrial oxidative phosphorylation to glycolysis despite the availability of oxygen, which is similar to the Warburg effect in cancer. Importantly, uric acid, a byproduct of fructose metabolism, likely plays a key role in favoring glycolysis by stimulating inflammation and suppressing aconitase in the tricarboxylic acid cycle. A consequent accumulation of glycolytic intermediates connects to the production of biosynthetic precursors, proteins, lipids, and nucleic acids, to meet the increased energy demand for the local inflammation. Here, we discuss the possibility of fructose and uric acid may mediate a metabolic switch toward glycolysis in CKD. We also suggest that sodium-glucose cotransporter 2 (SGLT2) inhibitors may slow the progression of CKD by reducing intrarenal glucose, and subsequently fructose levels.

## Introduction

Chronic kidney disease (CKD) has increased in the last decades and is a major cause of morbidity and mortality. Central to both diabetic and non-diabetic CKD is intrarenal inflammation and fibrosis. Here we present a novel hypothesis that fructose, either provided in the diet or produced endogenously, could play a key role in causing disease through its ability to induce inflammation through a Warburg effect. We also posit that this could explain the protective benefit of the sodium-glucose cotransporter-2 (SGLT2) inhibitors. While others have suggested that SGLT2 inhibitors may provide renal protection by reversing the Warburg effect (1), here we suggest endogenous fructose metabolism could be the mediator of the Warburg effect in this manuscript and we suggest a mechanism by which SGLT2 inhibitors could reduce fructose metabolism in the kidney. Since fructose is endogenously produced even in non-diabetic conditions, our hypothesis could be applied to how SGLT2 inhibitors improve both diabetic and non-diabetic CKD.

## Fructose, the Metabolic Syndrome, and CKD

Fructose is a simple sugar present in fruit and honey, and is also a major component of added sugars such as sucrose (a disaccharide of fructose and glucose) and high fructose corn syrup (HFCS, a combination of monosaccharide of fructose and glucose). Fructose intake has skyrocketed over the last century in association with the overall increased intake of added sugars.

**Figure 1:**
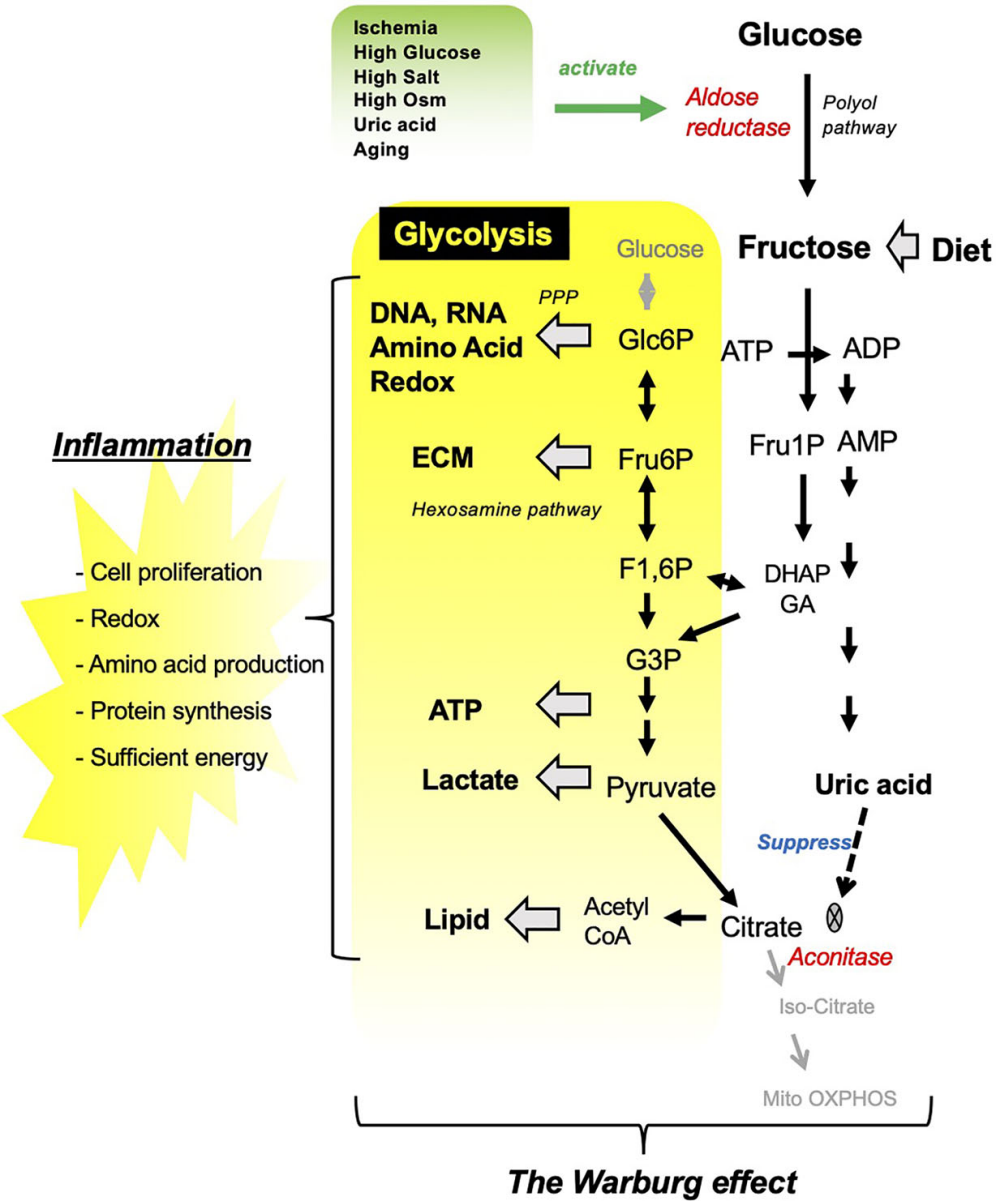
Fructose metabolism resembles the Warburg effect. Several pathological conditions stimulate aldose reductase, which converts glucose into fructose in the kidney. Fructose either from diet or from endogenous system under pathological condition is converted by fructokinase into Fructose 1-phosphate, which channels into glycolytic pathway. In turn, fructose metabolism results in uric acid production as a by-product, which suppress TCA cycle by inhibiting aconitase. ECM, extra cellular matrix.

Fructose can also be produced in the body by activation of the aldose reductase (AR) in the polyol pathway (**Figure 1**). A variety of stimuli are known to increase AR expression, including ischemia, hypoxia, hyperglycemia, hyperosmolality, and uric acid (2–5). While endogenous fructose production is usually low, there is increasing evidence that endogenous fructose production is increased not only in diabetes (6, 7), but also from a high carbohydrate diet, salty foods, and alcohol common to the western diet (8–11).

Research has implicated a role for fructose in many noncommunicable diseases, including obesity, diabetes, nonalcoholic fatty liver disease, and heart disease (12, 13) and both acute and chronic kidney disease (5, 6, 14, 15). Classically, this has been ascribed to fructose’s effect to stimulate oxidative stress, endothelial dysfunction, stimulation of vasopressin, and uric acid generation (12, 13, 16).

Recently we reviewed evidence that fructose may also aid cancer growth by turning on a metabolic switch favoring mitochondrial respiration over glycolysis, resembling the Warburg effect (17, 18). The Warburg effect is also likely involved in the progression of non-tumor disorders, including pulmonary hypertension, cardiovascular diseases, neuronal disorders, and kidney diseases (19). Here we suggest that the Warburg effect due to fructose might have a role in chronic kidney disease (CKD).

## Fructose Metabolism and the Warburg effect

Glycolysis is the metabolic pathway that converts glucose into pyruvate, that can enter the tricarboxylic acid (TCA) cycle in mitochondria where ATP is generated through oxidative phosphorylation. Fructose is distinct from glucose in that it is uniquely metabolized to Fructose 1-phosphate (Fru1P). Fru1P can be subsequently metabolized to link with the glycolytic pathway (**Figure 1**). During fructose metabolism, the activation of the C isoform of fructokinase (Ketohexokinase-C; KHK-C) reduces both phosphate and adenosine triphosphate (ATP) in the cell, and triggers the degradation of adenosine monophosphate (AMP) by AMP deaminase toward uric acid production. Uric acid is an intracellular pro-oxidant and is capable of suppressing aconitase, the enzyme catalyzing citrate to isocitrate in the TCA cycle. As a result, fructose can act as a metabolic switch favoring more rapid energy generation from glycolysis compared to energy generated by mitochondrial respiration despite the availability of oxygen. Similar to the Warburg effect in cancer growth, activated glycolysis supplies several intermediates linking to subsequent metabolic pathways, including pentose phosphate pathway, hexosamine pathway, and lipid synthesis, and these biosynthetic precursors contribute to the inflammatory reaction (17).

## Mechanisms by Which Fructose Stimulates Kidney Inflammation

**Figure 2:**
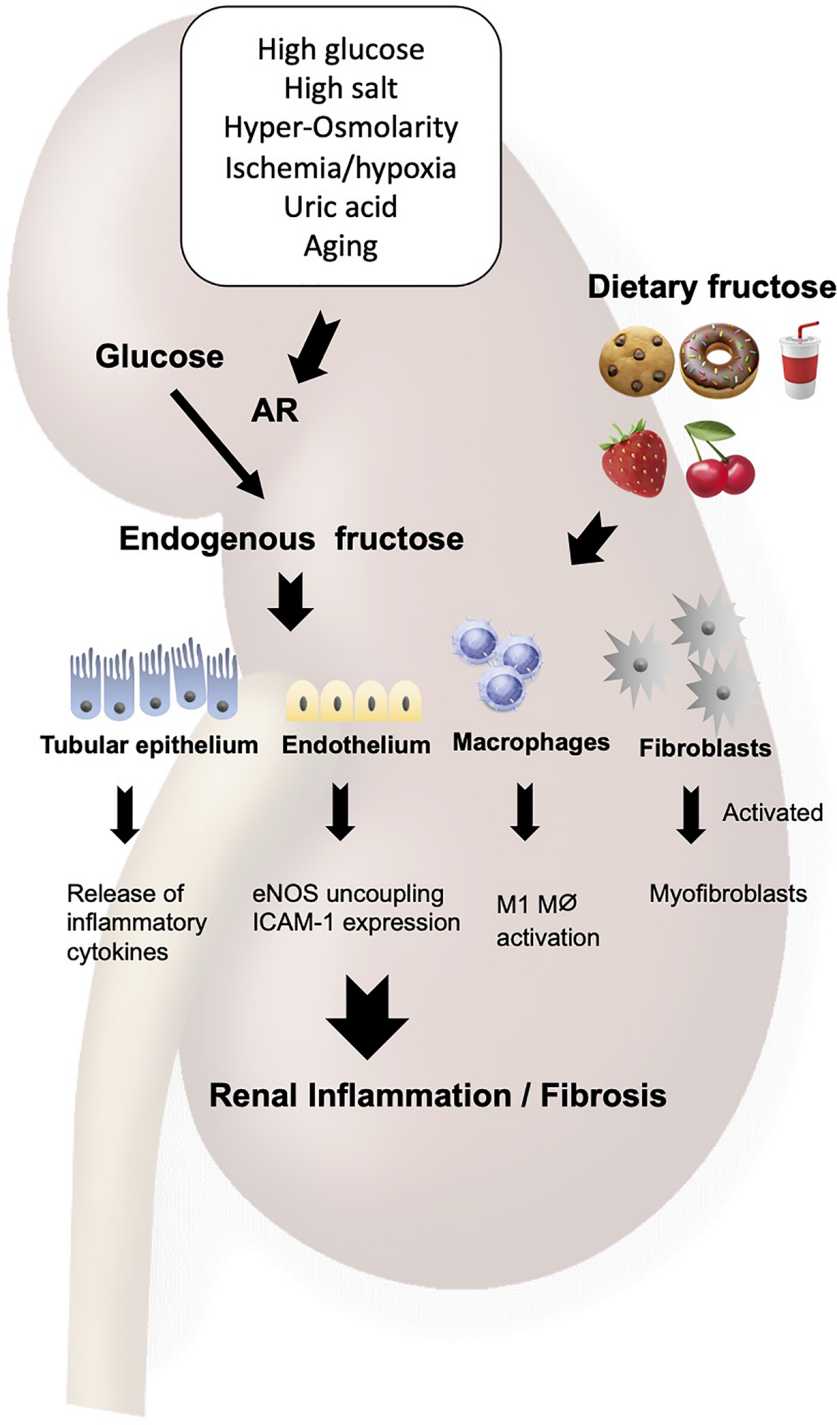
Fructose induces renal inflammation. Fructose either from diet or from endogenous system under pathological condition acts on the tubular epithelial cells, endothelial cells, macrophages and fibroblasts to cause the Warburg effect, leading to inflammation and fibrosis in the kidney. AR, aldose reductase; ICAM-1, intercellular adhesion molecule-1; eNOS, endothelial NO synthase.

In the kidney, dietary fructose is completely filtered through glomerulus, and reabsorbed in the proximal tubular epithelial cells through fructose transporters expressed in the apical membrane. The fructose is physiologically utilized in the cytosol as a substrate for gluconeogenesis to maintain systemic glucose concentration (18). The kidney is capable of producing fructose endogenously to cope with several pathological conditions. For example, ischemia, high glucose, and high osmolarity, all of which are key components of CKD, stimulate aldose reductase and activates the polyol pathway (**Figure 2**).

Excessive amount of dietary fructose is deleterious in the kidney. In fact, normal rats develop mild tubulointerstitial inflammation and fibrosis on high fructose diet. The tubular epithelial vimentin expression, collagen III deposition, and immune cell infiltration were induced by dietary fructose in rodents (15, 20). In the pre-existing kidney injury, fructose accelerates the progression of renal injuries with prominent inflammatory changes in both glomeruli and tubulointerstitium (14). Cultured proximal tubular cells were found to release inflammatory cytokines, including monocyte chemoattractant protein-1 (MCP-1) in response to fructose, a metabolic response mediated by uric acid (21).

The inflammatory response to fructose is induced not only by dietary fructose but also by the endogenous production of fructose in the tubular epithelial cells. In fact, mouse studies demonstrated that diabetes or hypoxia render the renal tubular epithelial cells to release several inflammatory cytokines, including NFκB activation, IL6 and CCL2 expressions, all of which were blunted in fructokinase-deficient mice (5, 6).

Endothelial cells are also stimulated to release intercellular adhesion molecule-1 (ICAM-1) in response to fructose (22). A likely mechanism is an ability of fructose to reduce nitric oxide (NO) availability in the endothelial cells as NO donors mitigated the fructose-induced ICAM-1 expression. This effect was shown to be due to the uncoupling of the endothelial NO synthase (eNOS) resulting from fructose-induced oxidative stress (23–25). Fructose-induced generation of uric acid could be also involved in this process as uric acid directly impairs endothelial function (16, 26, 27).

## CKD is Associated With Worsening Renal Hypoxia

Under normal physiological conditions, the kidney medulla is under a low oxygen condition with the partial oxygen pressure in the range of 10 to 20 mmHg, contrasting with that in the cortex which is about 50 mmHg (28). Physiological hypoxia largely depends on high demand of oxygen by renal tubular cells to maintain electrolyte transport. Oxygen supply is also constrained in this area because the vascular system is operated with a countercurrent system, in which oxygen diffuses from arterial to venous vasa recta and leaves the outer medulla deficient in oxygen (28). Under pathological conditions, a low oxygen level is further accelerated. For example, the loss of glomerular capillaries in glomerular sclerosis decreases the blood flow to the distal peritubular capillaries with further reduction in oxygen supply. Similarly, CKD-associated anemia can lower oxygen supply, while constriction of the efferent arteriole by intrarenal activation of the renin angiotensin aldosterone system reduces blood flow to tubulointerstitial area with similar effects on oxygen delivery (28). Since these are likely shared mechanisms in the progression of kidney diseases, hypoxia is considered as unifying pathway toward end-stage kidney disease (28, 29).

The kidneys are physiologically equipped with compensatory responses to hypoxia. Protection systems include the activation of hypoxia-induced factor-1α (HIF-1α), which is capable of stimulating the expression of erythropoietin for increasing erythrocytes, and the induction of vascular endothelial growth factor (VEGF) for inducing angiogenesis (30), both of which help deliver oxygen to hypoxic peripheral tissues. However, these compensatory reactions could turn to be deleterious under several pathological conditions. In particular, HIF could turn to be profibrotic under sustained hypoxia in CKD (31). The mechanism is likely involved in the ability of HIF to favor glycolysis over mitochondrial respiration, and to induce the production of endogenous fructose (32, 33). Nevertheless, in the setting of chronic hypoxia, it is likely that mitochondrial function may be progressively reduced, so that a switch for glycolysis is needed.

## The Warburg Effect Is Involved in CKD

The mitochondria has long been recognized as a site of increased oxidative stress in diabetes, and aberrant activation of mitochondria could also play a key role in diabetic complications (34–36), although there remains some controversy (37). However, recent evidence has shown that mitochondrial function is rather suppressed in diabetes, and restoration of normal mitochondrial health improves renal, cardiovascular, and neuronal outcomes (38–42). Consistently with these findings, experimental studies have shown that glycolytic intermediates and enzymes are upregulated in the kidney cortex in type 2 diabetes (42). Similarly, metabolites in mitochondrial citrate cycle were significantly reduced in patients with diabetic nephropathy compared to healthy controls (43). These data suggest that activated glycolysis is dominant over mitochondrial function and plays a pathological role in diabetic nephropathy.

There is also evidence that there may be a shift from oxidative stress to glycolysis in other types of CKD. One example is Autosomal dominant polycystic kidney disease (ADPKD), which is caused by loss-of-function mutations in either *PDK1* or *PKD2* (44). Rowe et al. found that cultured mouse embryonic fibroblasts (MEFs) derived from the *Pkd^-/-^* mice preferentially utilized higher amount of glucose, but excreted higher amount of lactate into culture medium than cells from wild type mice (45). In addition, *Pkd^-/-^* MEFs produced higher ATP content, which were associated with the upregulation of glycolysis enzymes and had only a minor effect by oligomycin, an inhibitor of mitochondrial ATP synthesis, suggesting that ATP is produced by glycolysis, but not by mitochondrial respiration. Likewise, the mouse lacking *Pkd* in the renal tubules, as a mouse model for ADPKD, exhibited glycolysis activation while blocking glycolysis with 2DG, a glucose analog, succeeded to attenuate tubular cell proliferation, leading to the reductions in kidney size and cyst formation (45, 46).

A shift to glycolysis has also been observed in a model of unilateral ureteral obstruction and in a TGF-β1-treated renal fibrosis model. Specifically, Ding et al. found that myofibroblast activation in the kidneys was associated with enhanced glucose uptake and lactate production in the kidneys that could be attenuated by blocking glycolysis by 2-Deoxy glucose treatment. It was then shown that this represented a TGF-β1-dependent metabolic switch favoring glycolysis over mitochondrial respiration. These data suggest that the Warburg effect could play a key role in the process of renal fibrosis (47).

## Fructose as a Mechanism for Inducing the Warburg Effect in CKD

The observation that CKD is associated with worsening intrarenal ischemia and hypoxia could have major effects on intra-renal metabolism. As we mentioned, hypoxia-associated HIF-1α stimulates endogenous fructose production and metabolism. Park et al. studied the role of fructose with the naked mole rats, which can survive longer time under hypoxic condition, and found that a mechanism for the tolerance to hypoxia is attributed to their capability to endogenously produce fructose (32). Fructose can be metabolized even under a low oxygen condition while it can provide several biosynthetic intermediates through several pathways to meet the demand for cell protection (as discussed in above section).

However, while fructose was likely meant to be protective in the setting of ischemia, under pathological conditions fructose may have deleterious consequences. Mirtschink et al. found that fructokinase was upregulated under a low oxygen condition as a HIF target gene, but it contributed to the development of the hypertrophic heart in mice while cardiac hypertrophy was blocked in fructokinase deficient mice (33). In the kidneys, endogenous fructose could be deleterious in several pathological conditions. Andres-Hernando et al. showed that a transient ischemia was capable of inducing endogenous fructose in the renal tubules, and again it was found to be deleterious as blocking fructose metabolism ameliorates the kidney injury in an ischemia-reperfusion mouse model (5).

Another setting where endogenous fructose production in the kidney is high is in diabetic nephropathy. In diabetic nephropathy there is not only intrarenal ischemia and hypoxia, but high trafficking of glucose in the proximal tubules. The local elevations in glucose are another major stimulus for fructose production. As fructokinase is present in proximal tubules (S1 to S3), it is likely that endogenous fructose production is high (7). Indeed, blocking fructokinase was found to be protective in experimental diabetic nephropathy (6).

The proximal tubular cells normally prefer lipids over glucose for energy production, so glycolysis has not been operated in this cell type. It would be accounted for by unbalance of enzymatic activations for glycolysis over those for gluconeogenesis (18). Since the proximal tubular cells are the major site of fructose metabolism in the kidney as this is where fructokinase is predominantly expressed, fructose metabolism physiologically links with gluconeogenesis, but not with glycolysis (18). However, this is not the case for damaged tubules. In fact, the damaged proximal tubular cells are often associated with mitochondrial alteration, leading to metabolic switch from mitochondrial oxidative phosphorylation to glycolysis with the amplified expression of glycolytic enzymes (48). Importantly, when fructose is metabolized with glucose, glucokinase activity is enhanced (49–52).

## Could the Beneficial Effects of SGLT2 Inhibitors in Diabetic and Non-diabetic CKD be Due to Prevention of Metabolic Switch Towards Glycolysis?

SGLT2 inhibitors have recently received an attention from clinicians and researchers for their major therapeutic benefits that extend beyond its glycemic control in both non-diabetic and diabetic kidney diseases and in the cardiovascular complications associated to CKD (53, 54). While the precise mechanisms remain unclear, recent studies have indicated that the protective effects might be accounted for by the prevention of the metabolic switch form lipid oxidation to glycolysis as aberrant glycolysis was likely associated with epithelial-to-mesenchymal transformation of proximal tubule cells in diabetic nephropathy (55, 56). In addition, SGLT2 inhibitors may reduce intra-renal work by blocking glucose uptake, and thereby reducing intra-renal hypoxia with the blocking of HIF-1α accumulation, and with the preventing a reduction of klotho, events that are expected to reduce glycolysis (57, 58). An additional protective effect exerted by SGLT2 inhibition is to chronically shift the fuel utilization toward fatty substrates to induce a significant increment in lipolysis and ketogenesis (59). The increase in ketone content also suggests an increase in β-oxidation and a reduction in the rate of glycolysis (60), which may explain both cardioprotective and nephroprotective effects (61). The stimulation of AMPK and sirtuin-1 is likely another mechanism for the protective effect of SGLT2 inhibitors (62).

One of the main actions of SGLT2 inhibitors is to block absorption of glucose into the S1 and S2 segments of the proximal tubule, and this should act to reduce the amount of glucose converted to fructose. Since some fructokinase is expressed at the site (15), this could represent a way to block the Warburg effect. Consistent with this suggestion, blocking fructokinase reduces the severity of diabetic nephropathy in mice (6, 7). However, we have previously suggested that the blocking of glucose uptake into the S1 and S2 segments of the proximal tubule might increase the amount of glucose reabsorbed by the S3 segment, which could lead to sufficient fructose generation that its metabolism by fructokinase could result in tubular injury and acute kidney injury (63). In the overall balance, however, the use of SGLT2 inhibitors would be expected to be protective for the kidney.

## Controversial Role for Glycolysis vs. Oxidative Metabolism in Macrophage Activation

Macrophages are involved in fructose-induced renal inflammation (14, 15, 22). Two major macrophage phenotypes exist: a pro-inflammatory (M1) phenotype that relies on glycolysis and an anti-inflammatory/pro-resolving (M2) phenotype that depends on oxidative phosphorylation (64, 65). Since macrophages express Glut5 on their surface (66), and fructose stimulates macrophages to release pro-inflammatory cytokines (67, 68), fructose may be an ideal fuel for the M1 macrophage due to its ability to stimulate glycolysis. Although several studies indicate that mitochondrial respiratory chain is also active in inflammatory M1 macrophages, it may function to produce reactive oxygen species to kill infectious bacteria as opposed to stimulate ATP synthesis (69).

In contrast, a recent study demonstrated that oxidative metabolism, but not glycolysis, plays a dominant role of macrophage activation in fructose-induced inflammation since blocking oxidative phosphorylation, but not inhibition of glycolysis, suppressed the release of pro-inflammatory cytokines (67). A key finding was that fructose stimulates glutamine uptake to activate TCA cycle, leading to mTORC1 activation for the release of inflammatory cytokines in human monocytes and mouse macrophages. While fructose metabolism inhibits aconitase, and therefore suppresses TCA cycle, glutamine metabolism supplies α-ketoglutarate that can bypass this step allowing oxidative phosphorylation to occur (70, 71).

Given these facts, macrophages likely utilize either glycolysis or oxidative phosphorylation for their activation, and the precise mechanism as to how macrophages select metabolic pathways remains unclear. A potential explanation is that oxygen availability is a determinant as the activity of cytochrome c oxidase activity decreases when the oxygen concentration drops below 1.0mM (72). Likewise, Semba et al. also recently examined the role of oxygen in macrophage migration and showed that in severe hypoxia, glycolysis is dominant while cytochrome c oxidase activity is severely blocked (73). In turn, cytochrome c activity turns on with oxygen availability, and glycolysis is completely replaced for oxidative phosphorylation under aerobic condition.

Taken together, these studies suggest that macrophages depend on both glycolysis or oxidative phosphorylation for their activation, and the selection of metabolic pathways may partially depend on the oxygen availability. It is likely that glycolysis drives M1 macrophage activation under hypoxic condition, whereas oxidative phosphorylation is used under aerobic conditions (73). These studies suggest that fructose metabolism could also be affected by oxygen availability, so that the activation of metabolic pathway in macrophage might be determined by both the fructose concentration and oxygen levels.

## Conclusions

Our studies suggest fructose may play a role in CKD. This could occur secondary to excessive intake of dietary fructose from the diet. However, it might be also a consequence of endogenous fructose production driven by intrarenal ischemia or increased glucose trafficking. Finally, the suppression of these pathways may explain the protective effect of SGLT2 inhibitors in both diabetic and non-diabetic CKD.

## Author Contributions

TN designed the story of manuscript and wrote entire manuscript. RJ and LS-L significantly edited the manuscript. AA-H, BR-I, PB, HK, MK, and ML edited the part of their own research area. All authors contributed to the article and approved the submitted version.

## Funding

Japan Society for the Promotion of Science (21K10284).

## Conflict of Interest

ML, LS-L and RJ have equity in a start-up company developing fructokinase inhibitors (Colorado Research Partners LLC). TN and RJ also have equity with XORTX therapeutics which is developing novel xanthine oxidase inhibitors. RJ is also a consultant for Horizon Pharmaceuticals, Inc. BR-I is a recipient of the Cátedra Salvador Zubirán, Universidad Nacional de México and Instituto Nacional de Ciencias Médicas y Nutrición “Salvador Zubirán, Ciudad de México, Mexico. PB has acted as a consultant for AstraZeneca, Bayer, Bristol-Myers Squibb, Boehringer Ingelheim, Eli-Lilly, Sanofi, Novo Nordisk, and Horizon Pharma. PB serves on the advisory boards of AstraZeneca, Boehringer Ingelheim, Novo Nordisk and XORTX.

The remaining authors declare that the research was conducted in the absence of any commercial or financial relationships that could be construed as a potential conflict of interest.
